# Supplementary material for: A Particle-Based COVID-19 Simulator With Contact Tracing and Testing
Source: IEEE Open J Eng Med Biol. 2021 Mar 8;2:111–7. doi: 10.1109/OJEMB.2021.3064506 (PMC8545033; doi:10.1109/OJEMB.2021.3064506)
Supplement: Supplementary file 1 — Simulation results for different values of the maximum velocity in the supplementary materials. [file supp1-3064506.pdf]

A Particle-Based COVID-19 Simulator with Contact Tracing and Testing

Askat Kuzdeuov, *Member, IEEE* Aknur Karabay, Daulet Baimukashev, Bauyrzhan Ibragimov, and Huseyin Atakan Varol*, *Senior Member, IEEE*

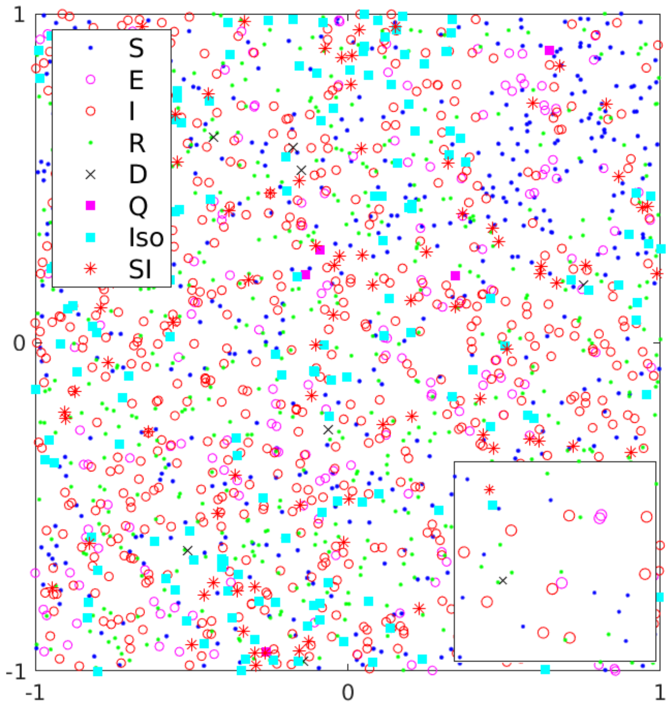

Fig. 1. Visualization of the particles on the map including their epidemic states.

I. TRANSITIONS BETWEEN EPIDEMIC STATES

In this simulator, particles move on a 2D map as shown in Fig. 1. The vector $\boldsymbol{e} \in \mathbb{R}^n$ stores the epidemic states of all n particles, where each element e_i contains the epidemic status of the particle i, $1 \leq i \leq n$, and has one of these values based on the epidemic status: 0 - Susceptible, 1 - Exposed, 2 - Infected, 3 - Recovered, 4 - Dead, 5 - True Quarantined, 6 - True Isolated, 7 - Severely Infected, 8 - False Isolated, and 9 - False Quarantined. Transitions between epidemic states are shown in Fig. 2. The parameters of the model are given in Table I.

At the beginning of the simulation, all n particles are in the Susceptible state ($e_i = 0$ for $i = 1,...,n$). Then, we randomly choose n_e particles and change their epidemic status to the Exposed ($e_{r_k} = 1$, for $k = 1,...,n_e$, where $\boldsymbol{r} \in \mathbb{R}^{n_e}$ is a vector of pseudorandom integers drawn from the discrete uniform distribution on the interval [1, n]). Then, all particles start moving on the map and come into contact with each other.

A susceptible particle becomes exposed when it comes into contact with a contagious particle (i.e., Exposed, Infected, True Quarantined, True Isolated, or Severely Infected). A contact between two particles is assumed to happen when the distance between them is less than x_{thr}. In order to find the contacted susceptible particles, first, we need to identify contagious particles in the epidemic state vector $\boldsymbol{e}$. Thus, we define $\boldsymbol{\kappa_C} \in \mathbb{R}^n$ that contains the indices of contagious particles, where $\kappa_{C_i} = 1$ for the contagious particle, otherwise $\kappa_{C_i} = 0$. Similarly, we define vectors that contain indices of the Susceptible ($\boldsymbol{\kappa_S} \in \mathbb{R}^n$), Exposed ($\boldsymbol{\kappa_E} \in \mathbb{R}^n$), Infected ($\boldsymbol{\kappa_I} \in \mathbb{R}^n$), True Quarantined ($\boldsymbol{\kappa_{TQ}} \in \mathbb{R}^n$), True Isolated ($\boldsymbol{\kappa_{TI}} \in \mathbb{R}^n$), and Severely Infected particles ($\boldsymbol{\kappa_{SI}} \in \mathbb{R}^n$). The $\boldsymbol{\kappa_C}$ is calculated based on the disease transmission probabilities as

$$\boldsymbol{\kappa_C} = \boldsymbol{\kappa_I} | (\boldsymbol{\kappa_E} \odot (\boldsymbol{r} < \epsilon_{exp})) | (\boldsymbol{\kappa_{TQ}} \odot (\boldsymbol{r} < \epsilon_{qua})) | (\boldsymbol{\kappa_{TI}} \odot (\boldsymbol{r} < \epsilon_{qua})) | (\boldsymbol{\kappa_{SI}} \odot (\boldsymbol{r} < \epsilon_{sev})) \quad (1)$$

where $\boldsymbol{r} \in \mathbb{R}^n$ is a vector of uniformly distributed random numbers in the range [0, 1], $\odot$ is a pointwise multiplication operator and | is a logical OR operator.

Then, we identify the current positions of contagious particles on the map as

$$P_{C_j} = P_i \text{ if } \kappa^c_{S_i} = 1 \text{ for } i = 1,\dots,n,\ j = 1,\dots m. \quad (2)$$

where m is a number of non-zero elements in $\boldsymbol{\kappa_C}$, $\boldsymbol{P} \in \mathbb{R}^{nx2}$ is a matrix that stores the current positions of all particles. Then, we employ the `rangesearch`(P, P_C, x_{thr}) function [1] of MATLAB to create a vector $\boldsymbol{\kappa_A} \in \mathbb{R}^n$ that stores indices of particles that were in contact with the contagious particles. This

TABLE I. List of simulation parameters and their descriptions.

Parameter	Description
n	Total number of particles
n_e	Initial number of exposed particles
T	Simulation length in days
x_{thr}	Minimum distance to transmit the disease
v_{max}	Maximum allowed speed of particles
λ	Speed gain
sir	Daily rate of Infected/Isolated particles getting Severely Infected
γ_{mor}	Severely Infected to Dead transition probability
ϵ_{exp}	Transmission probability of Exposed
ϵ_{qua}	Transmission probability of Quarantined
ϵ_{sev}	Transmission probability of Severely Infected
t_{exp}	Exposure period in days
t_{inf}	Infection period in days
β	Ratio of the population using a contact tracing app
θ	Number of daily tests per thousand people
sn	Sensitivity of tests
sp	Specificity of tests

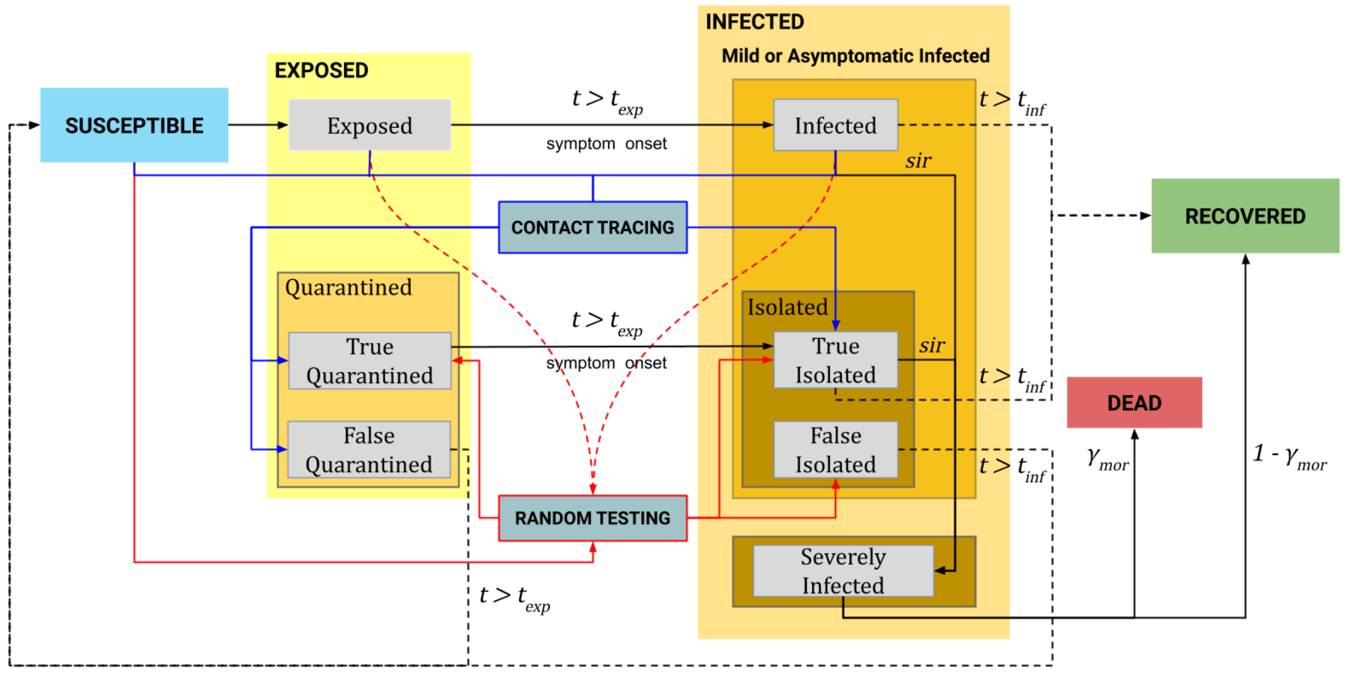

Fig. 2. The statechart of the particle-based SEIR epidemic simulator.

way, we can extract the indices of the contacted susceptible particles from $\boldsymbol{\kappa_A}$ as

$$\boldsymbol{\kappa_S^c} = \boldsymbol{\kappa_A} \ \& \ \boldsymbol{\kappa_S} \tag{3}$$

Then, we change their epidemic state to Exposed and reset their epidemic time in the susceptible state:

$$e_i = \begin{cases} 1, & \text{if } \kappa_{S_i}^c = 1 \\ e_i, & \text{otherwise,} \end{cases} \qquad t_i = \begin{cases} 0, & \text{if } \kappa_{S_i}^c = 1 \\ t_i, & \text{otherwise.} \end{cases} \tag{4}$$

The exposed particles whose epidemic time in this state exceeds the exposure period t_{exp} move to the Infected state, and their epidemic time is set to zero as

$$e_i = \begin{cases} 2, & \text{if } e_i = 1 \ \& \ t_i \geq t_{exp} \\ e_i, & \text{otherwise,} \end{cases} \qquad t_i = \begin{cases} 0, & \text{if } e_i = 1 \ \& \ t_i \geq t_{exp} \\ t_i, & \text{otherwise.} \end{cases} \tag{5}$$

Some portion of the infected particles are selected randomly and moved to the Severely Infected state during their infection period t_{inf}, and regarded as positively-tested (i.e., hospitalized)

$$e_i = \begin{cases} 7, & \text{if } e_i = 7 \ \& \ r_i \leq sir \cdot \Delta t \\ e_i, & \text{otherwise} \end{cases} \qquad ts_i = \begin{cases} \text{d}, & \text{if } e_i = 7 \ \& \ r_i \leq sir \cdot \Delta t \\ ts_i, & \text{otherwise} \end{cases} \tag{6}$$

where $\boldsymbol{r} \in \mathbb{R}^n$ is a vector of uniformly distributed random numbers in the range [0, 1]; $\boldsymbol{ts} \in \mathbb{R}^n$ stores for each particle the date when its COVID-19 test result was positive, and d is the current date.

Then, the infected particles move to the Recovered state after t_{inf} days:

$$e_i = \begin{cases} 3, & \text{if } e_i = 2 \ \& \ t_i \geq t_{inf} \\ e_i, & \text{otherwise.} \end{cases} \tag{7}$$

Severely infected particles die with γ_{mor} probability after t_{inf} days while the rest move to the Recovered state:

$$e_i = \begin{cases} 4, & \text{if } e_i = 7 \ \& \ t_i \geq t_{inf} \ \& \ r_i \leq \gamma_{mor} \\ 3, & \text{if } e_i = 7 \ \& \ t_i \geq t_{inf} \ \& \ r_i > \gamma_{mor} \\ e_i, & \text{otherwise.} \end{cases} \tag{8}$$

The testing module randomly tests particles in the Susceptible, Exposed, and Infected states that were not tested before. The results of the tests might be true positive or false positive because of the test sensitivity sn and specificity sp:

$$ts_i = \begin{cases} \text{d}, & \text{if } (e_i = 1 \mid e_i = 2) \ \& \ ts_i = 0 \ \& \ r_i \leq \theta sn \Delta t \\ \text{d}, & \text{if } e_i = 0 \ \& \ ts_i = 0 \ \& \ r_i \leq \theta (1 - sp) \Delta t \\ ts_i, & \text{otherwise.} \end{cases} \tag{9}$$

where d is the current date.

Particles that were correctly detected by the test are moved to the True Quarantined and True Isolated states as

$$e_i = \begin{cases} 5, & \text{if } e_i = 1 \ \& \ ts_i = d \ \& \ r_i \leq \theta sn \Delta t \\ 6, & \text{if } e_i = 2 \ \& \ ts_i = d \ \& \ r_i \leq \theta sn \Delta t \\ e_i, & \text{otherwise.} \end{cases} \tag{10}$$

Susceptible particles that were incorrectly tested positive

move to the False Isolated state:

$$e_i = \begin{cases} 8, & \text{if } e_i = 0 \ \& \ ts_i = d \ \& \ r_i \le \theta(1-sp)\Delta t \\ e_i, & \text{otherwise.} \end{cases} \tag{11}$$

Then, the contact tracing module checks the contacts of the positive tested particles. The contacts of the exposed particles transition to the True Quarantined state, while the contacts of the Infected particles move to the True Isolated state. Similarly, the contacts of the Susceptible particles, which were incorrectly detected by the test, are sent to the False Quarantined state.

Particles in the True Quarantined state transition to the True Isolated when their epidemic time exceeds the exposure period t_{exp}:

$$e_i = \begin{cases} 6, & \text{if } e_i = 5 \ \& \ t_i \ge t_{exp} \\ e_i, & \text{otherwise,} \end{cases} \quad t_i = \begin{cases} 0, & \text{if } e_i = 5 \ \& \ t_i \ge t_{exp} \\ t_i, & \text{otherwise.} \end{cases} \tag{12}$$

On the other hand, particles in the False Quarantined state return to the Susceptible state when the exposure period t_{exp} ends:

$$e_i = \begin{cases} 0, & \text{if } e_i = 9 \ \& \ t_i \ge t_{exp} \\ e_i, & \text{otherwise,} \end{cases} \quad t_i = \begin{cases} 0, & \text{if } e_i = 9 \ \& \ t_i \ge t_{exp} \\ t_i, & \text{otherwise.} \end{cases} \tag{13}$$

Some portion of the particles in the True Isolated state are selected randomly and moved to the Severely Infected state during their infection period t_{inf}:

$$e_i = \begin{cases} 7, & \text{if } e_i = 6 \ \& \ r_i \le sir \cdot \Delta t \\ e_i, & \text{otherwise,} \end{cases} \tag{14}$$

The other particles in the True Isolated state recover after t_{inf} days:

$$e_i = \begin{cases} 3, & \text{if } e_i = 6 \ \& \ t_i \ge t_{inf} \\ e_i, & \text{otherwise.} \end{cases} \tag{15}$$

On the other hand, particles in the False Isolated state go back to the Susceptible state when the infection period t_{inf} ends:

$$e_i = \begin{cases} 0, & \text{if } e_i = 8 \ \& \ t_i \ge t_{inf} \\ e_i, & \text{otherwise,} \end{cases} \quad t_i = \begin{cases} 0, & \text{if } e_i = 8 \ \& \ t_i \ge t_{inf} \\ t_i, & \text{otherwise.} \end{cases} \tag{16}$$

II. Impact of the Particle Velocity on the Spread of the Epidemic

The velocity of the particles plays an important role in the spread of the epidemic among the population. Higher values of v_{max} lead to a faster spread of the disease. In order to show this, we conducted simulations with different values of $v_{max} = \{0.5, 0.75, 1.0, 1.25, 1.5\}$. The total number of particles n is 10,000, and the initial number of exposed particles n_e is 10. The other parameters of the simulation are shown in Table II.

TABLE II. Parameters of the simulation.

n	n_e	x_{thr}	ϵ_{exp}	ϵ_{qua}	ϵ_{sev}	t_{exp}
10,000	10	0.0001	0.7	0.3	0.3	5
t_{inf}	sir	γ_{mor}	β	θ	sn	sp
14	0.02	0.15	0	0	0	0

For each considered maximum velocity v_{max}, we ran 10 simulations and took averaged results. The averaged results for all cases are summarized in Fig. 3. According to the results, the epidemic reaches the peak faster with the increased speed of particles. In addition, the number of exposed and infected particles at the peak also grows with increased velocity. As a result, the numbers of severely infected, recovered, and dead particles increase faster. The results show that a higher velocity results in a higher number of contacts per unit time. Therefore, we changed the value of v_{max}, according to the epidemic timeline, to imitate different levels of the quarantine in Lecco.

III. Particle-based SEIR Simulation the Canton of Geneva

To validate our particle-based simulator and model for the province of Lecco, we have performed simulations for the canton of Geneva in Switzerland. The official statistics [2] for this country have been shared by its government since February 25, 2020 and a number of seroprevalence studies have been performed.

The canton of Geneva is one of the most heavily hit regions of Switzerland by the COVID-19 epidemic, with 42,159 officially registered cases by January 12, 2021. However, according to the seroprevalence study [3] in the canton of Geneva from April 6 to June 30, 2020, around 7.8% of the population (38,595) had antibodies, which is seven times greater than the officially confirmed cases at that date (5,412). In order to adjust the parameters of the model for Geneva, we relied on the total cases that were corrected using the seroprevalence survey results and officially confirmed number of deaths. The total number of particles was set to 499,480 (the population of the canton) [4]. The initial number of exposed particles was set to 10. We started the simulation on January 5, 2020, in accordance with the official statistics [2]. To initialize the number of tests per thousand people, we used the averaged value 0.5 from the official statistics [2]. We adjusted parameters, such as sir and γ_{mor} while the other parameters were the same as for the province of Lecco. All parameters of the simulation are provided in Table IV. To imitate the lockdown in Geneva, we decreased the maximum speed of particles v_{max} and λ, in accordance with the epidemic timeline (see Fig. 4). The detailed timeline of policies in the canton of Geneva is provided in Table III. We used these to adjust parameters and calibrate the model for this specific region.

We ran 10 simulations, and the averaged results are shown in Fig. 4. The results show that by tuning only a few parameters of the calibrated model we can fit data for other countries. The model also predicted the peak of the epidemic accurately, because, according to the official data, the number of cases increased starting from the middle of March and remained high

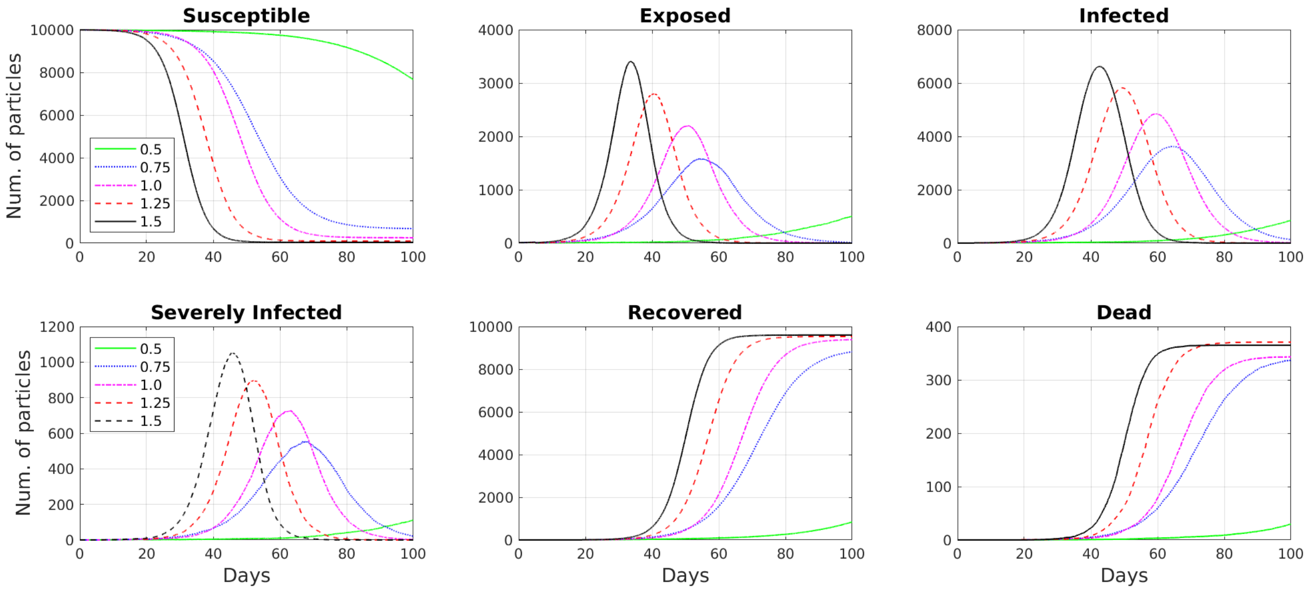

Fig. 3. Simulation results for different values of v_{max}.

TABLE III. COVID-19 policies timeline in the canton of Geneva [5]

Day	Date	Event
0	5/1/2020	Start of the simulation.
50	25/2/2020	The COVID-19 data repository was launched [3]. First confirmed case.
70	16/3/2020	Lockdown in the canton.
111	26/4/2020	1st stage of lockdown easing. Hospitals for all purposes are open.
127	11/5/2020	2nd stage of lockdown easing Shops, markets are open.
155	8/6/2020	3rd stage of lockdown easing Schools are open.
200	19/7/2020	End of the simulation.

TABLE IV. Simulation Parameters for the canton of Geneva.

v_{max} (Days)	λ (Days)	x_{thr} (Days)	ϵ_{exp}	ϵ_{qua}	ϵ_{sev}	t_{exp}
0.02 (0-50)<br>0.009 [50-70)<br>0.003 [70-111)<br>0.004 [111-127)<br>0.06 [127-155)<br>0.02 [155-200)	0.002 (0-50)<br>0.0009 [50-70)<br>0.0003 [70-111)<br>0.0004 [111-127)<br>0.006 [127-155)<br>0.02 [155-200)	7.5e-5 (0-155)<br>6e-5 [155-200)	0.7	0.3	0.3	5
t_{inf}	sir	γ_{mor}	β	θ	sn	sp
14	0.01	0.08	0	0.5	0.95	0.99

until the middle of April. In addition, the model predicted the second wave of the epidemic after 180 days. The same can be observed in the official data (see Fig. 5).

References

[1] MATLAB. (2020) Rangesearch. Last accessed on 2020-10-2: https://www.mathworks.com/help/stats/rangesearch.html#mw_51f765d0-dc9e-42f3-83dd-472717aa3f57.

[2] S. Amt. (2021) Data COVID-19 Swiss Cantons and Principality of Liechtenstein. Last accessed on 2021-01-15: https://github.com/openZH/covid_19/tree/master/fallzahlen_kanton_total_csv9.

[3] A. Richard, A. Wisniak, J. Perez-Saez, H. Garrison-Desany, D. Petrovic, G. Piumatti *et al.*, "Seroprevalence of anti-SARS-CoV-2 IgG antibodies, risk factors for infection and associated symptoms in Geneva, Switzerland: a population-based study," *medRxiv*, 2020. [Online]. Available: https://www.medrxiv.org/content/early/2020/12/18/2020.12.16.20248180

[4] "Canton of geneva." [Online]. Available: https://datacommons.org/place/nuts/CH013

[5] "Switzerland to start easing covid-19 restrictions from april 27," Apr 2020. [Online]. Available: https://www.cnbc.com/2020/04/16/switzerland-to-start-easing-covid-19-restrictions-from-april-27.html

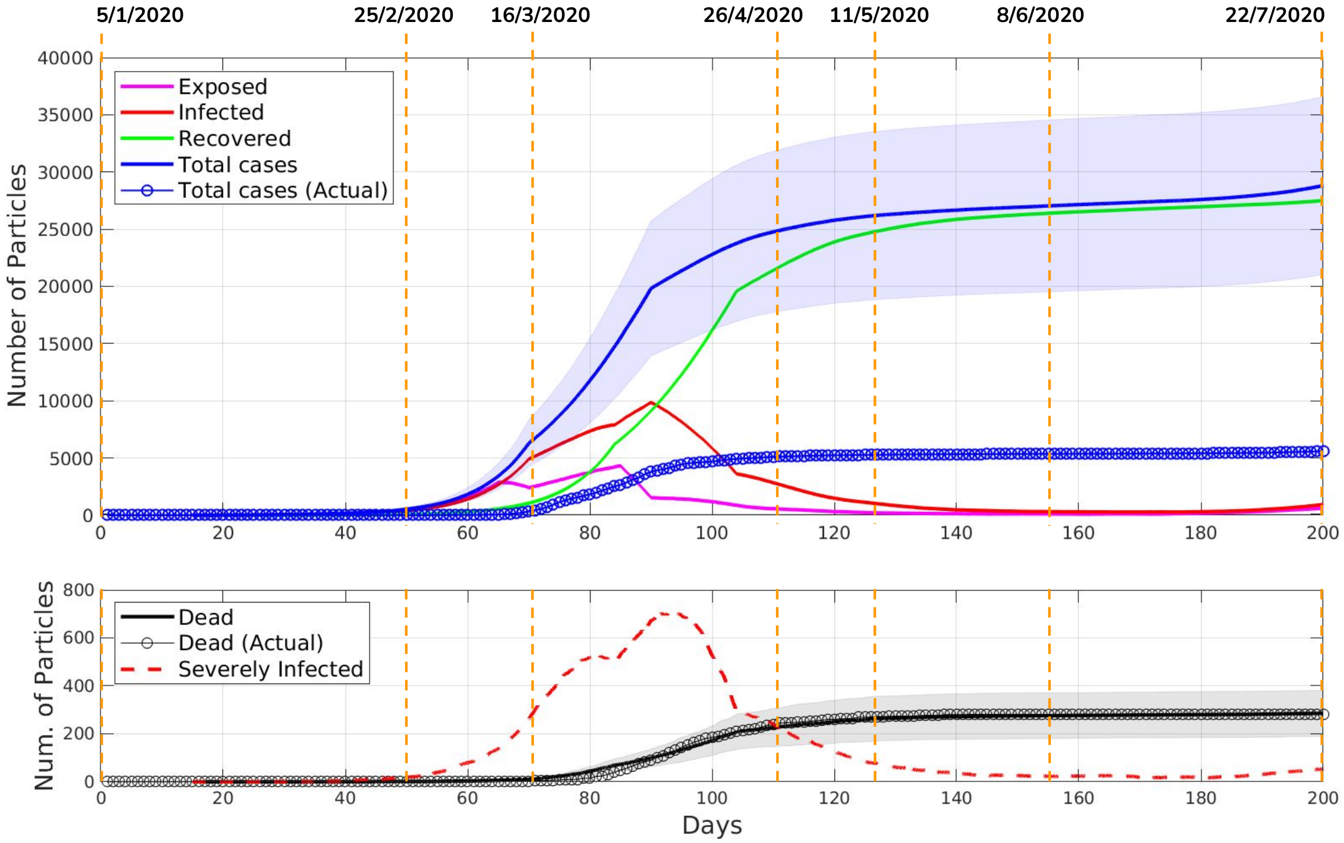

Fig. 4. The averaged results of ten simulations for the canton of Geneva. The upper plot shows the states of the epidemic simulation versus time. The dates of important NPIs are shown by vertical dashed lines and listed in Table III. One standard deviation around the average Total case curve is shaded in blue. The bottom plot compares the average number of deaths in the simulation with the actual number of deaths due to COVID-19. This plot also shows the simulation results for the number of severely infected particles as well. One standard deviation around the average Dead state curve is shaded in grey.

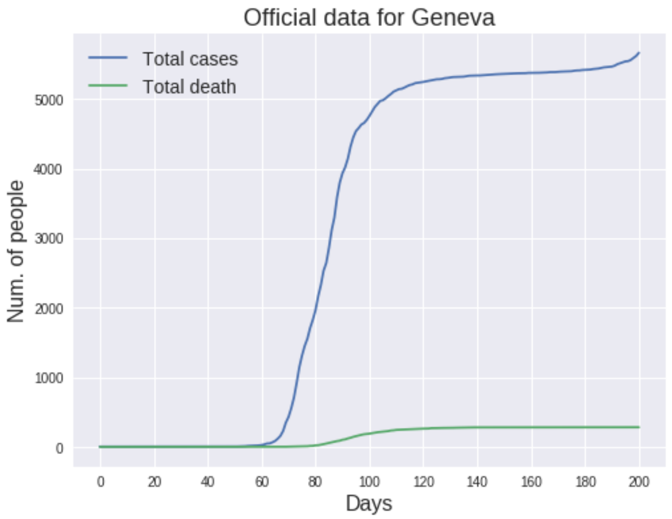

Fig. 5. Actual COVID-19 data for Geneva from 5 January 2020 to 11 August 2020.
